# Resveratrol as a Modulator of Adriamycin-, Taxol-, and Cisplatin-Induced Cytotoxicity in MCF-7 Breast Cancer Cells

**DOI:** 10.3390/ijms27072979

**Published:** 2026-03-25

**Authors:** Burcu Biltekin, Hafize Uzun, Ayhan Bilir

**Affiliations:** 1 Department of Histology and Embryology, Faculty of Medicine, Istanbul Atlas University, Istanbul 34403, Turkey; ayhan.bilir@atlas.edu.tr; 2 Department of Biochemistry, Faculty of Medicine, Istanbul Atlas University, Istanbul 34403, Turkey; huzun59@hotmail.com

**Keywords:** resveratrol, breast cancer, MCF-7, doxorubicin, paclitaxel, cisplatin, apoptosis

## Abstract

Breast cancer (BC) remains the most diagnosed malignancy among women worldwide, with approximately 2.3 million new cases and over 670,000 deaths reported annually. Resistance to conventional chemotherapeutic agents and treatment-related toxicity remain major challenges in BC management. Resveratrol, a naturally occurring polyphenol, has been proposed as a potential modulator of chemotherapy response; however, comparative evidence regarding its interaction with different classes of chemotherapeutic agents is limited. This study aimed to comparatively assess the effects of resveratrol on the cytotoxic, antiproliferative, and apoptotic responses induced by adriamycin, taxol, and cisplatin in MCF-7 BC cells. MCF-7 cells were treated with adriamycin, taxol, cisplatin, and resveratrol, either alone or in combination, across multiple concentrations for 24, 48, 72, and 96 h. Cell viability was evaluated using the trypan blue exclusion assay. Cellular proliferation was assessed via BrdU incorporation, while apoptosis and cell death profiles were analyzed using Annexin V staining and flow cytometry. Exposure to individual chemotherapeutic agents induced a significant time- and dose-dependent reduction in MCF-7 cell viability (*p* < 0.001). Resveratrol co-treatment further modulated chemotherapy-induced cytotoxicity in an agent- and time-dependent manner. Combination treatments markedly suppressed DNA synthesis compared with single-agent exposure (*p* < 0.01) and significantly increased apoptotic cell populations. Flow cytometric Annexin V/PI analysis demonstrated that early apoptotic cells ranged from 3.2–11.3% in single-agent treatments and increased to 0.04–50.4% in resveratrol-based combination groups. Similarly, late apoptotic/secondary necrotic cell fractions increased from 4.1–4.4% in single-agent treatments to 2.1–69.9% following combination therapy, indicating a substantially higher overall cell death response in selected treatment conditions. This study demonstrates that resveratrol modulates the cytotoxic and apoptotic responses of MCF-7 BC cells to adriamycin, taxol, and cisplatin in an agent- and time-dependent manner. The findings indicate that resveratrol acts as a context-dependent modulator of chemotherapy response. Although resveratrol generally enhanced cytotoxic and apoptotic responses in combination treatments, the magnitude of these effects varied depending on the chemotherapeutic agent and exposure conditions. Further preclinical and clinical studies are warranted to define their therapeutic relevance.

## 1. Introduction

Breast cancer (BC) remains the most frequently diagnosed cancer among women worldwide and represents a major cause of cancer-related mortality. According to recent global cancer statistics, approximately 2.3 million new BC cases are diagnosed annually, accounting for nearly 1 in 4 cancers among women, with over 670,000 deaths reported worldwide each year [1]. These figures underscore the substantial global burden of BC and highlight the need for improved therapeutic approaches and optimized treatment strategies [1,2]. Current treatment strategies rely predominantly on cytotoxic chemotherapy, including anthracyclines such as doxorubicin (Adriamycin), taxanes such as paclitaxel (Taxol), and platinum-based agents such as cisplatin. Despite their central role in standard BC regimens, the clinical utility of these agents is often constrained by dose-limiting toxicities and the emergence of intrinsic or acquired chemoresistance [3,4,5].

Resveratrol (3,5,4′-trihydroxy-trans-stilbene) is a naturally occurring phytoalexin belonging to the phenylpropanoid family and is synthesized by plants in response to environmental stressors, including microbial infection and ultraviolet (UV) irradiation. Upon UV exposure, the biologically predominant trans isomer undergoes geometric conversion to the cis form. Although the cis isomer exhibits biological activity, it has been less extensively studied, largely due to its lower natural abundance. The photochemical behavior of resveratrol is mainly characterized by trans–cis isomerization and fluorescence, which are strongly influenced by environmental conditions [6,7,8,9,10,11,12].

Accumulating evidence suggests that resveratrol may function as a chemosensitizer capable of modulating the efficacy of conventional chemotherapeutic agents while potentially mitigating systemic toxicity. In vitro studies have shown that resveratrol enhances doxorubicin-induced cytotoxicity in BC cells by promoting apoptosis, increasing the proportion of annexin V^+^/PI^−^ and annexin V^+^/PI^+^ cell populations, suppressing multidrug resistance mechanisms, and altering intracellular drug accumulation [13]. In contrast, interactions between resveratrol and taxanes appear to be context-dependent, with both synergistic and antagonistic effects reported according to estrogen receptor status and downstream signaling pathways. Similarly, resveratrol has been reported to sensitize BC cells to cisplatin by impairing homologous recombination-mediated DNA repair, notably through downregulation of RAD51 expression [7,8,9].

The estrogen receptor-positive MCF-7 BC cell line is a well-established in vitro model of luminal-type BC and provides a relevant platform for investigating drug–drug interactions and differential cytotoxic responses [14]. Despite growing interest in resveratrol–chemotherapy combinations, comparative studies evaluating its modulatory effects across distinct classes of chemotherapeutic agents within a unified experimental framework remain limited.

Therefore, we hypothesized that resveratrol differentially modulates the cytotoxic and apoptotic responses induced by adriamycin, paclitaxel, and cisplatin in MCF-7 BC cells. To our knowledge, this study is the first to systematically evaluate the modulatory role of resveratrol in Adriamycin-, Taxol-, and cisplatin-induced cytotoxicity in MCF-7 BC cells. Accordingly, the present study aimed to comparatively evaluate the effects of resveratrol on chemotherapy-induced cytotoxicity, proliferation, and apoptosis, with particular emphasis on Annexin V^+^/PI-defined cell death profiles, to identify potential synergistic or antagonistic interactions that may inform rational combination strategies for BC treatment.

## 2. Results

### 2.1. Effects of Resveratrol and Chemotherapeutic Agents on MCF-7 Cell Viability

**Figure 1:**
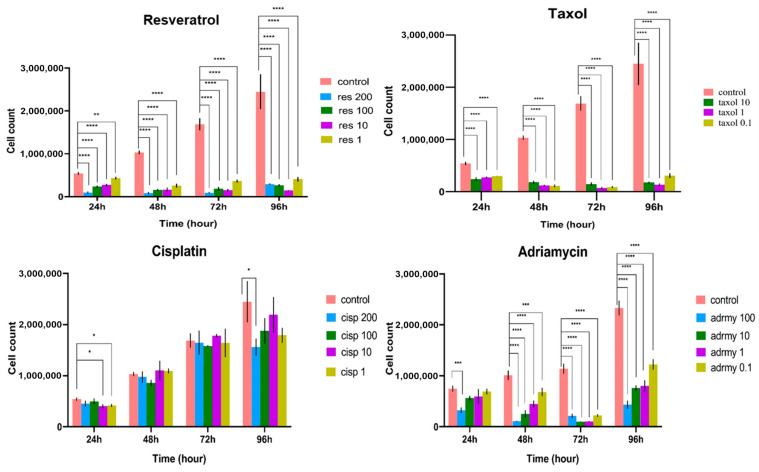
Effects of single agents on MCF-7 cell viability. MCF-7 cells were treated with resveratrol (1–200 μM), cisplatin (1–200 μM), adriamycin (0.1–100 μM), or taxol (paclitaxel; 0.1–10 μg/mL) for 24, 48, 72, and 96 h. Cell viability was determined using the trypan blue exclusion assay. Viable cells were counted from independent cultures at each time point and compared with time-matched controls. Data is expressed as the percentage of viable cells relative to control. * *p* < 0.05; ** *p* < 0.01; *** *p* < 0.001, **** *p* < 0.001, all of them are vs. control.

**Figure 2:**
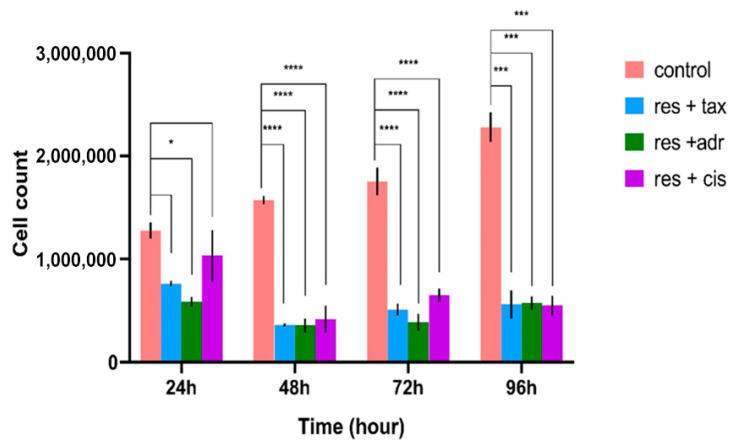
Effects of resveratrol–chemotherapeutic drug combinations on MCF-7 cell viability. MCF-7 cells were treated with resveratrol in combination with taxol, cisplatin, or adriamycin at selected representative concentrations (resveratrol: 100 µM; cisplatin: 100 µM; adriamycin: 10 µM; taxol: 1 µg/mL) for 24, 48, 72, and 96 h. These concentrations were chosen based on the dose–response profiles obtained in Figure 1, corresponding to submaximal cytotoxic levels to allow evaluation of combinatorial effects. Cell viability was determined using the trypan blue exclusion assay. Viable cells were counted from independent cultures at each time point and compared with time-matched controls. Data are expressed as the percentage of viable cells relative to control. * *p* < 0.05; *** *p* < 0.001, **** *p* < 0.001, all of them are vs. control.

The effects of resveratrol, Taxol, cisplatin, Adriamycin, and their respective combinations on MCF-7 cell viability were evaluated using the trypan blue exclusion assay at 24, 48, 72, and 96 h. As shown in Figure 1, treatment with individual chemotherapeutic agents resulted in a time-dependent reduction in cell viability compared with untreated controls. Resveratrol alone induced a moderate but significant decrease in viable cell percentage over time. For combination experiments, representative concentrations corresponding to submaximal cytotoxic levels (resveratrol: 100 µM; cisplatin: 100 µM; adriamycin: 10 µM; taxol: 1 µg/mL) were selected based on the dose–response data obtained from single-agent treatments. Notably, combined treatments of resveratrol with Taxol, cisplatin, or Adriamycin produced a more pronounced reduction in cell viability at all time points, with the greatest inhibitory effect observed at 72 and 96 h (Figure 2).

### 2.2. Inhibition of DNA Synthesis and Cell Proliferation

**Figure 3:**
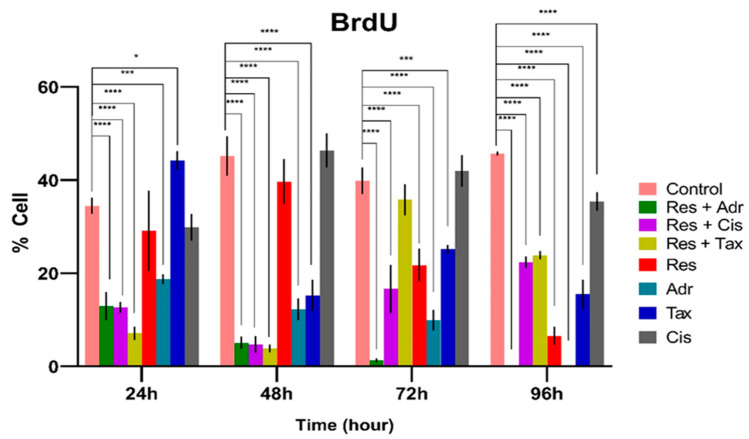
Time-dependent inhibition of DNA synthesis by resveratrol and chemotherapeutic combinations. MCF-7 cells were treated with resveratrol and chemotherapeutic agents either alone or in combination using the following concentrations: resveratrol (100 µM), cisplatin (100 µM), adriamycin (10 µM), and taxol (1 µg/mL). Cells were incubated for up to 96 h. Proliferation rates were determined using the BrdU labeling index at 24, 48, 72, and 96 h. Each bar represents the percentage of BrdU-positive cells relative to the total population. * *p* < 0.05; *** *p* < 0.001, **** *p* < 0.001, all of them are vs. control.

Cell proliferation was assessed based on BrdU incorporation following exposure to individual agents and their combinations for up to 96 h. As illustrated in Figure 3, BrdU labeling indices declined progressively in treated cells compared with controls, indicating suppressed DNA synthesis. While resveratrol or chemotherapeutic agents alone reduced proliferation in a time-dependent manner, combination treatments resulted in significantly lower BrdU-positive cell percentages, particularly at later time points. These findings demonstrate enhanced inhibition of DNA synthesis with resveratrol-based combination therapies.

### 2.3. Flow Cytometric Analysis of Cell Viability and Cell Death

**Figure 4:**
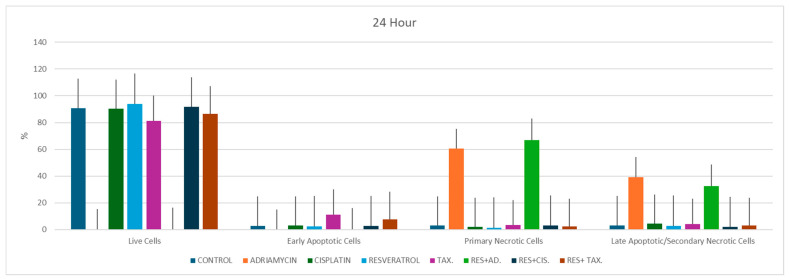
Flow cytometric analysis of cell viability and cell death following treatment. MCF-7 cells were treated for 24 h with resveratrol and chemotherapeutic agents either alone or in combination at the following concentrations: resveratrol (100 µM), cisplatin (100 µM), adriamycin (10 µM), and taxol (1 µg/mL). Apoptosis and cell death were analyzed using Annexin V-FITC/PI staining. Cell populations were classified as viable, early apoptotic, primary necrotic, and late apoptotic/secondary necrotic based on fluorescence profiles.

Flow cytometric Annexin V/PI analysis performed at 24 h revealed marked alterations in cell viability and cell death profiles following treatment (Figure 4). Untreated control cells exhibited a high proportion of viable cells (90.86%), with minimal early apoptotic (2.82%), primary necrotic (3.06%), and late apoptotic/secondary necrotic populations (3.27%) [values normalized to ensure total population equals 100%].

Treatment with individual chemotherapeutic agents produced heterogeneous cytotoxic responses. Adriamycin caused a pronounced loss of viability, with viable cells decreasing to 0.24%, accompanied by a marked increase in primary necrotic cells (60.44%) and late apoptotic/secondary necrotic cells (39.32%). In contrast, cisplatin-treated cells maintained a high proportion of viable cells (90.41%), with relatively low levels of apoptosis (early apoptosis: 3.2%; late apoptosis/secondary necrosis: 4.41%). Paclitaxel treatment produced a moderate apoptotic response, increasing early apoptotic cells to 11.26% while maintaining 81.3% viable cells. Resveratrol alone had a limited cytotoxic effect, with most cells remaining viable (93.69%) and only modest levels of apoptosis observed (early apoptosis: 2.36%; late apoptosis/secondary necrosis: 2.72%).

The resveratrol–taxol combination demonstrated a predominantly viable cell population (86.6%) with relatively low levels of early apoptosis (7.8%), primary necrosis (2.5%), and late apoptotic/secondary necrotic cells (3.1%), indicating a comparatively limited cytotoxic effect under these conditions. In contrast, the resveratrol–adriamycin combination induced extensive cytotoxicity, characterized by a marked predominance of primary necrosis (66.92%), accompanied by late apoptotic/secondary necrotic cells (32.71%), and minimal viable cell fraction (0.33%), reflecting severe loss of membrane integrity. The resveratrol–cisplatin combination showed a relatively limited cytotoxic effect, with 91.71% viable cells, and low proportions of early apoptotic (2.86%), primary necrotic (3.29%), and late apoptotic/secondary necrotic cells (2.14%).

Collectively, these findings indicate that resveratrol modulates chemotherapy-induced cell death in a treatment-specific manner, with distinct patterns of apoptosis and necrosis depending on the chemotherapeutic agent used. Notably, the observed differences reflect shifts in the relative distribution of mutually exclusive cell populations rather than overlapping percentages. The predominance of primary necrosis in the resveratrol–adriamycin group suggests rapid cytotoxic injury, whereas the relatively preserved viability in the resveratrol–taxol and resveratrol–cisplatin groups indicates a more limited or delayed cytotoxic response at the evaluated time point.

### 2.4. Cell Cycle Distribution Following Treatment

**Figure 5:**
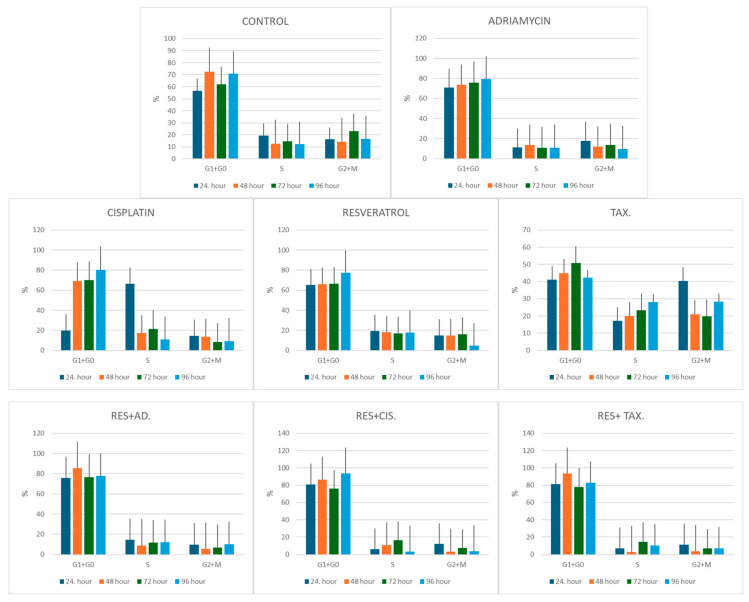
Treatment-specific effects of resveratrol and chemotherapeutic agents on cell cycle distribution in MCF-7 cells. MCF-7 cells were treated with individual agents and their respective combinations using the following concentrations: resveratrol (100 µM), cisplatin (100 µM), adriamycin (10 µM), and taxol (1 µg/mL) for 24, 48, 72, and 96 h. The percentage of cells in the G_0_/G_1_, S, and G_2_/M phases was determined via flow cytometric analysis of DNA content. The figure presents the distribution of cell cycle phases for each treatment group at the indicated time points, enabling direct comparison of treatment-specific effects.

**Figure 6:**
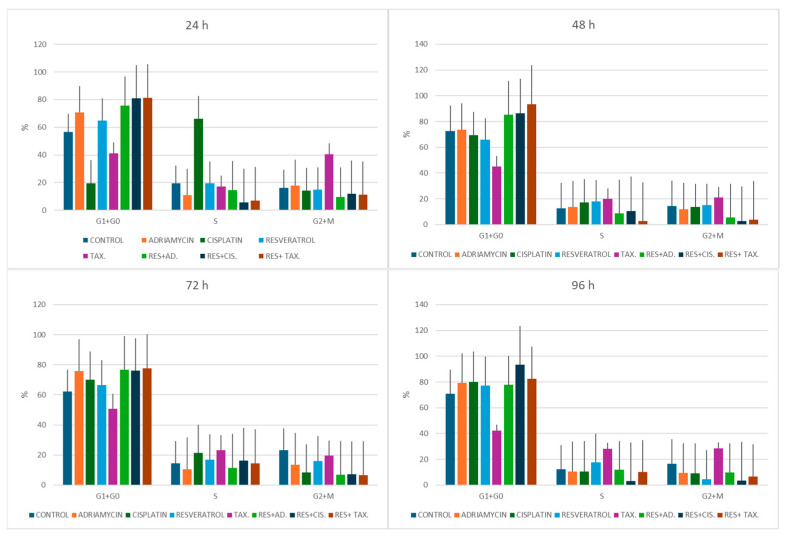
Time-dependent modulation of cell cycle progression following treatment with resveratrol-based combinations. MCF-7 cells were treated with resveratrol, chemotherapeutic agents (Adriamycin, Cisplatin, and Taxol), and their respective combinations using the following concentrations: resveratrol (100 µM), cisplatin (100 µM), adriamycin (10 µM), and taxol (1 µg/mL). Cell cycle distribution (G_0_/G_1_, S, and G_2_/M) was determined via flow cytometry at 24, 48, 72, and 96 h. The figure illustrates the temporal changes in cell cycle phase distribution across the 96-h treatment period.

The effects of resveratrol and chemotherapeutic agents on cell cycle progression were evaluated via flow cytometry at 24, 48, 72, and 96 h following treatment with selected representative concentrations of the tested agents (Figure 5 and Figure 6). Control cells demonstrated a relatively stable distribution across the G_0_/G_1_, S, and G_2_/M phases throughout the experimental period. At 24 h, treatment with individual chemotherapeutic agents produced distinct alterations in cell cycle distribution. Adriamycin treatment increased the G_0_/G_1_ population to 70.8%, while cisplatin markedly elevated the S-phase fraction to 66.2%, indicating replication-associated stress. Paclitaxel treatment resulted in accumulation in the G_2_/M phase (40.5%), consistent with its known microtubule-stabilizing mechanism that interferes with mitotic progression. Resveratrol alone produced moderate cell cycle changes, with a slight increase in G_0_/G_1_-phase cells (65%) compared with controls.

Combination treatments induced more pronounced cell cycle alterations. The resveratrol–adriamycin combination increased the G_0_/G_1_ fraction to 75.6%, whereas the resveratrol–cisplatin and resveratrol–taxol combinations produced a strong G_0_/G_1_ accumulation (80.9% and 81.4%, respectively), accompanied by a marked reduction in S-phase cells.

At 48 h, these changes became more evident. Control cells showed a typical distribution with 72.7% in G_0_/G_1_, whereas resveratrol-based combinations produced a substantial accumulation of cells in G_0_/G_1_ phase (85.4% for resveratrol–adriamycin, 86.5% for resveratrol–cisplatin, and 93.5% for resveratrol–taxol), suggesting progressive inhibition of DNA synthesis and cell cycle progression.

At 72 h, similar trends persisted. Combination-treated groups maintained elevated G_0_/G_1_ fractions (76.6–77.7%) compared with control cells (62.1%), while S-phase proportions remained relatively reduced in most treatment groups.

By 96 h, control cells maintained a balanced cell cycle distribution (70.8% G_0_/G_1_), whereas resveratrol–cisplatin and resveratrol–taxol combinations exhibited pronounced G_0_/G_1_ accumulation (93.4% and 82.6%, respectively) and markedly reduced S-phase fractions. Paclitaxel alone continued to show elevated G_2_/M fractions (28.4%), consistent with mitotic arrest.

## 3. Discussion

The present study provides comparative evidence that resveratrol differentially modulates the cytotoxic, antiproliferative, and apoptotic effects of distinct classes of chemotherapeutic agents in estrogen receptor-positive MCF-7 BC cells. Our findings demonstrate that while adriamycin, paclitaxel, and cisplatin each induced a significant, time- and dose-dependent reduction in cell viability, co-treatment with resveratrol resulted in agent-specific alterations in chemotherapy-induced cytotoxicity. Notably, resveratrol-based combinations were associated with a more pronounced suppression of DNA synthesis, as evidenced by reduced BrdU incorporation, and with enhanced apoptosis characterized by increased Annexin V^+^/PI^−^ and Annexin V^+^/PI^+^ cell populations in selected treatment groups. Collectively, these results indicate that the biological effects of resveratrol are highly context-dependent and influenced by the chemotherapeutic backbone, highlighting its role as a selective modulator of treatment response rather than a uniform chemosensitizer.

### 3.1. Agent- and Time-Dependent Effects of Resveratrol on Chemotherapy-Induced Cytotoxicity

In the present study, adriamycin (doxorubicin), paclitaxel, and cisplatin administered as single agents induced a significant time- and dose-dependent reduction in MCF-7 cell viability, consistent with their established cytotoxic profiles in estrogen receptor-positive BC models [15,16,17]. Importantly, co-treatment with resveratrol modulated chemotherapy-induced cytotoxicity in an agent- and exposure time-dependent manner. This finding is in line with previous reports indicating that resveratrol does not act as a uniform chemosensitizer but rather exerts context-dependent effects influenced by the chemotherapeutic backbone and cellular environment [18,19,20,21]. Several studies have demonstrated that resveratrol can enhance doxorubicin-induced cytotoxicity in MCF-7 cells, whereas its interaction with paclitaxel has been reported to vary from synergistic to antagonistic depending on estrogen receptor signaling and intracellular context [18,22,23,24,25,26]. Similarly, resveratrol-mediated modulation of cisplatin sensitivity has been described in BC models, supporting the heterogeneity observed in our viability data [7,22,23,24,25,26,27,28].

### 3.2. Resveratrol Potentiates Chemotherapy-Induced Suppression of DNA Synthesis

Consistent with the viability findings, BrdU incorporation assays demonstrated a more pronounced suppression of DNA synthesis in resveratrol–chemotherapy combination groups compared with single-agent treatments. Resveratrol has previously been shown to inhibit proliferative activity in MCF-7 cells by interfering with growth-related signaling pathways and cell cycle progression [29,30,31,32,33]. Moreover, enhanced antiproliferative effects of doxorubicin in the presence of resveratrol have been reported, supporting a cooperative interaction on DNA synthesis and cellular proliferation [8,13]. The stronger inhibition observed at early time points (24–48 h) may reflect an enhanced initial antiproliferative response to the combination treatment. At later time points (72–96 h), the intrinsic cytotoxic effects of the chemotherapeutic agents alone may predominate, reducing the apparent combinatorial advantage. Our BrdU data corroborate these findings and further indicate that resveratrol may potentiate the antiproliferative effects of selected chemotherapeutic agents under specific experimental conditions.

### 3.3. Agent-Specific Modulation of Apoptosis by Resveratrol in Combination with Chemotherapy

Annexin V/PI staining and flow cytometric analyses revealed marked alterations in apoptotic and necrotic cell populations following treatment. In the present study, resveratrol co-treatment modulated chemotherapy-induced cell death in an agent-dependent manner in MCF-7 cells. Early apoptotic cell fractions ranged from 3.2 to 11.3% in single-agent treatments. In contrast to the initially overestimated values, resveratrol-based combinations did not uniformly increase apoptotic responses; rather, distinct patterns were observed depending on the chemotherapeutic agent. Notably, the resveratrol–paclitaxel combination demonstrated a predominantly viable cell population with only modest apoptotic and necrotic fractions, indicating a relatively limited cytotoxic effect under the evaluated conditions. Similarly, late apoptotic/secondary necrotic cell fractions were relatively low in single-agent treatments (4.1–4.4%) and remained modest in the resveratrol–paclitaxel and resveratrol–cisplatin groups, while a marked increase was observed primarily in the resveratrol–adriamycin combination, which was characterized by extensive necrotic cell death. These findings suggest that resveratrol does not uniformly potentiate chemotherapy-induced apoptosis but instead exerts agent-specific modulatory effects, which may include enhancement or attenuation depending on the treatment context. This observation is consistent with previous studies reporting the pro-apoptotic and chemosensitizing properties of resveratrol in breast cancer models.

Previous in vitro studies have reported that resveratrol enhances doxorubicin-induced apoptosis in MCF-7 cells, reflected by increased Annexin V^+^/PI^−^ and Annexin V^+^/PI^+^ cell populations [8,28]. In the context of cisplatin, resveratrol has been shown to sensitize BC cells to apoptosis, potentially through impairment of homologous recombination-mediated DNA repair mechanisms, including downregulation of RAD51 [28,32,33,34]. Li et al. [35] demonstrated that resveratrol induces apoptosis in BC cells through metabolic modulation via inhibition of intracellular fatty acid synthase, as evidenced by increased Annexin V-positive cell populations. Consistent with these findings, our Annexin V/PI flow cytometric analyses revealed an elevated proportion of apoptotic cells in selected resveratrol–chemotherapy combinations in MCF-7 cells. However, unlike the direct pro-apoptotic effect reported by Li et al. [35], the apoptotic response observed in our study was agent- and context-dependent, suggesting that resveratrol primarily acts as an apoptotic sensitizer in the presence of specific chemotherapeutic agents. Together, these findings cell cycle 204 indicate that resveratrol-induced apoptosis may arise through distinct but complementary mechanisms depending on whether it is applied alone or in combination with cytotoxic therapies. The agent-specific increase in apoptotic fractions observed in our study is therefore consistent with existing literature and further supports the selective nature of resveratrol-mediated apoptotic modulation [18,36].

### 3.4. Resveratrol-Induced Modulation of Cell Cycle Arrest in Combination with Chemotherapy

Cell cycle analyses demonstrated that resveratrol, particularly in combination with specific chemotherapeutic agents, altered cell cycle distribution and promoted phase-specific cell cycle arrest. Previous reports have shown that resveratrol can induce G_0_/G_1_ or G_2_/M arrest in MCF-7 cells depending on concentration and exposure duration [37,38,39]. Enhanced cell cycle arrest has also been described in resveratrol–doxorubicin combination treatments, suggesting cooperative regulation of cell cycle checkpoints [8,40]. By contrast, interactions between resveratrol and paclitaxel have been reported to be highly context-dependent, which may explain the time-dependent and agent-specific cell cycle shifts observed in our experiments [10,41,42]. Ye et al. [42] reported that resveratrol exhibits higher anti-tumor efficacy in advanced BC organoids compared with several clinically relevant chemotherapeutic agents, highlighting its intrinsic anticancer potential in complex three-dimensional models. In line with these findings, our results demonstrate that resveratrol significantly enhances antiproliferative and apoptotic responses when combined with selected chemotherapeutic agents in MCF-7 cells, supporting the notion that resveratrol may be utilized as both a preventive and therapeutic agent in various cancers, including BC, by acting as a context-dependent modulator of therapeutic efficacy rather than a standalone cytotoxic compound [43,44]. Although cisplatin has frequently been reported to induce S-phase arrest in certain cancer cell models, cell cycle responses to cisplatin may vary depending on cell type and experimental conditions. In the present study, MCF-7 cells predominantly accumulated in the G0/G1 phase following cisplatin treatment, while the S-phase fraction remained relatively low. This observation may reflect activation of the G1/S checkpoint in response to cisplatin-induced DNA damage, preventing progression into the S phase. Similar variability in cisplatin-induced cell cycle arrest has been reported in previous studies [45,46].

### 3.5. Context-Dependent Effects of Resveratrol in Combination with Chemotherapeutic Agents

The biological effects of resveratrol in combination with chemotherapeutic agents appear to be context-dependent and influenced by the mechanism of action of the anticancer drug. The concentration ranges used in this study were selected based on biologically relevant exposure levels reported in previous in vitro studies, enabling the evaluation of drug-specific differences in apoptotic and antiproliferative responses following combination treatments.

Cisplatin + Resveratrol

The combination of resveratrol and cisplatin resulted in a marked increase in apoptotic cell populations compared with cisplatin treatment alone. Cisplatin is known to induce DNA damage through the formation of DNA crosslinks, which subsequently activates apoptotic signaling pathways. In the present study, however, the overall cytotoxic effect of the resveratrol–cisplatin combination remained limited, with a high proportion of viable cells, suggesting that the interaction may be relatively weak under the applied experimental conditions.

Adriamycin + Resveratrol

Adriamycin (doxorubicin) exerts its anticancer effects primarily through DNA intercalation and inhibition of topoisomerase II, leading to oxidative stress and apoptosis. In the present study, resveratrol co-treatment further enhanced the cytotoxic and antiproliferative effects of adriamycin in MCF-7 cells. Notably, this effect was characterized predominantly by necrotic cell death rather than classical apoptotic patterns, as reflected by the marked increase in primary necrotic cell populations and near-complete loss of viable cells. This finding suggests that the combination induces rapid and severe cellular injury, potentially overwhelming regulated apoptotic pathways. Doxorubicin-induced cytotoxicity is partly mediated by ROS generation. Given the antioxidant properties of resveratrol, its interaction with doxorubicin may modulate oxidative stress-related pathways in a context-dependent manner, potentially influencing apoptotic responses.

Paclitaxel + Resveratrol

Paclitaxel induces cytotoxicity mainly through microtubule stabilization and mitotic arrest. In contrast to an expected synergistic interaction, the resveratrol–paclitaxel combination in the present study demonstrated a relatively limited cytotoxic effect, with a predominance of viable cells and only modest apoptotic and necrotic fractions. This observation suggests that the interaction between resveratrol and paclitaxel may not be uniformly synergistic and may even attenuate paclitaxel-induced cytotoxicity under certain conditions. These findings are consistent with previous reports indicating that resveratrol can exert context-dependent or even antagonistic effects when combined with taxanes, depending on cellular signaling pathways and experimental conditions.

Overall, the modulatory effects of resveratrol observed in this study should be interpreted within a context-dependent framework. Resveratrol interacts with multiple intracellular signaling pathways involved in oxidative stress, apoptosis, and cell cycle regulation. Consequently, its influence on chemotherapy-induced cytotoxicity may vary depending on the mechanism of action of the chemotherapeutic agent, exposure concentration, and treatment duration. The present findings further emphasize that resveratrol does not act as a universal chemosensitizer but rather as a selective modulator of treatment response, with effects that may range from enhancement to attenuation depending on the therapeutic context.

### 3.6. Study Novelty and Contribution

A notable strength of the present study is the systematic comparison of resveratrol interactions with three widely used chemotherapeutic agents, adriamycin, paclitaxel, and cisplatin—within a single experimental framework. Unlike previous studies that typically focused on a single drug combination, this approach enabled the identification of drug-specific differences in cytotoxic, antiproliferative, and apoptotic responses. These findings provide novel insights into the context-dependent modulatory role of resveratrol in breast cancer chemotherapy.

### 3.7. Limitations

Several limitations of this study should be acknowledged. First, the findings are based on a single estrogen receptor-positive BC cell line, which may not fully capture the heterogeneity of BC subtypes. Second, the in vitro nature of the experimental model does not account for pharmacokinetic factors, tumor microenvironment interactions, or systemic metabolism that may influence resveratrol–chemotherapy interactions in vivo. Finally, while apoptotic and naturally occurring polyphenolic alterations were characterized at the cellular level, the underlying molecular mechanisms were not directly investigated. Accordingly, further mechanistic studies and in vivo validation are required to clarify the translational relevance of these observations.

### 3.8. Clinical Relevance

From a clinical perspective, the modulatory effects of resveratrol should be interpreted within a context-dependent framework. Resveratrol interacts with multiple intracellular signaling pathways involved in oxidative stress, apoptosis, and cell cycle regulation. Consequently, its influence on chemotherapy-induced cytotoxicity may vary according to the mechanism of action of the chemotherapeutic agent, drug concentration, and treatment duration. These factors may explain the drug-specific differences observed among cisplatin, adriamycin, and paclitaxel combinations in the present study.

The enhancement of antiproliferative and apoptotic responses observed in selected resveratrol–chemotherapy combinations supports the hypothesis that resveratrol may contribute to optimized combination strategies. Such interactions may potentially enable dose reduction or improved therapeutic tolerability of certain chemotherapeutic regimens. These findings provide a biological rationale for further preclinical and translational studies aimed at identifying the patient- and drug-specific contexts in which resveratrol supplementation could be clinically beneficial in BC therapy. However, further in vivo and clinical studies are required to confirm the translational relevance of these findings.

### 3.9. Future Research

Future studies should further investigate the molecular mechanisms underlying the context-dependent interactions between resveratrol and different classes of chemotherapeutic agents, with particular emphasis on metabolic regulation, DNA damage response, and cell cycle control. Moreover, extending these findings to BC organoid models will be critical to better recapitulate tumor heterogeneity and microenvironmental complexity, thereby improving the translational relevance of resveratrol-based combination strategies.

## 4. Materials and Methods

### 4.1. Drug Nomenclature

In this study, the chemotherapeutic agents adriamycin (doxorubicin hydrochloride; an anthracycline antibiotic), taxol (paclitaxel; a taxane-derived microtubule-stabilizing agent), and cisplatin (cis-diamminedichloroplatinum (II); a platinum-based compound) were used either alone or in combination with resveratrol (3,5,4′-trihydroxy-trans-stilbene). Resveratrol, a naturally occurring polyphenolic stilbene compound, was purchased from Sigma-Aldrich (Sigma Chemicals, St. Louis, MO, USA) and prepared according to the manufacturer’s instructions prior to experimental use.

Preparation of resveratrol: Resveratrol (98% purity) was dissolved in dimethyl sulfoxide (DMSO) to prepare a 1 mM stock solution. To ensure complete solubilization, the mixture was vortexed for 2 min. The resulting stock solution was divided into aliquots and stored at −20 °C, protected from light to prevent photochemical degradation. Immediately prior to experimental use, the stock was further diluted in the culture medium, ensuring that the final DMSO concentration did not exceed 0.1% (*v*/*v*) to mitigate vehicle-induced cytotoxicity.

### 4.2. MCF-7 Cell Culture

The human MCF-7 cell line was obtained from ATCC (MCF-7, ATCC^®^ HTB-22™, Manassas, VA, USA). Cells were maintained at 37 °C in a humidified atmosphere containing 5% CO_2_ in complete DMEM medium (DMEM; Gibco, Thermo Fisher Scientific, Waltham, MA, USA) supplemented with 10% heat-inactivated fetal bovine serum (FBS; Sigma-Aldrich, St. Louis, MO, USA), 2 mM L-glutamine, 100 U/mL penicillin, and 100 μg/mL streptomycin. Cells were passaged twice weekly, and all experiments were performed using semi-confluent cultures to ensure exponential growth conditions. For experimental treatments, MCF-7 cells were seeded into appropriate culture vessels and exposed to adriamycin (0.1–100 µM), paclitaxel (0.1–10 µg/mL), cisplatin (1–200 µM), and resveratrol (1–200 µM) either alone or in combination. Cells were incubated for 24, 48, 72, and 96 h to evaluate cell viability, proliferation, apoptosis, and cell cycle distribution. Untreated cells maintained under identical culture conditions served as the negative control group. Experimental groups included cells treated with resveratrol alone, individual chemotherapeutic agents alone (adriamycin, paclitaxel, or cisplatin), and their respective combination treatments. All experiments were performed in three independent biological replicates (n = 3). To minimize potential confounding effects related to nutrient depletion or accumulation of metabolic by-products during prolonged incubation, cells were seeded at appropriate densities to prevent excessive confluence. Control cultures maintained stable viability throughout the 24–96 h incubation period, indicating that the observed effects were attributable to the applied treatments rather than culture-related artifacts.

The concentration ranges were initially determined based on previously published literature and preliminary dose–response experiments. For combination studies, representative concentrations corresponding to submaximal cytotoxic levels were selected from these ranges (resveratrol: 100 µM; cisplatin: 100 µM; adriamycin: 10 µM; taxol: 1 µg/mL), allowing the assessment of drug–drug interactions without inducing excessive cell death. The use of submaximal concentrations in combination experiments enabled the detection of context-dependent modulatory effects of resveratrol, which may not be observable at highly cytotoxic doses.

### 4.3. Trypan Blue Exclusion Assay

Cell viability was assessed using the trypan blue exclusion assay, which distinguishes viable from non-viable cells based on membrane integrity. Following treatment with resveratrol, adriamycin, paclitaxel, and cisplatin either alone or in combination for 24, 48, 72, and 96 h, MCF-7 cells were harvested by trypsinization (0.25% trypsin–EDTA; Gibco, Thermo Fisher Scientific, USA) and centrifuged at 1000 rpm for 5 min. The resulting pellet was resuspended in phosphate-buffered saline (PBS). An aliquot of the cell suspension was mixed with an equal volume of 0.4% trypan blue solution (Sigma-Aldrich, St. Louis, MO, USA) and incubated for 2–3 min at room temperature. The mixture was then loaded onto a hemocytometer, and viable (unstained) and non-viable (blue-stained) cells were counted under an inverted microscope (Olympus BX51, Tokyo, Japan). Cell viability was calculated as the percentage of viable cells relative to the total number of cells counted [47].

All experiments were performed in three independent biological replicates (n = 3).

### 4.4. BrdU Cell Proliferation Assay

Cell proliferation was assessed by immunohistochemical detection of bromodeoxyuridine (BrdU) incorporation as an indicator of DNA synthesis, based on our previously published protocol [48] with minor modifications. Briefly, following treatment with the experimental agents for the indicated time periods, 20 µM of BrdU solution (BrdU; Sigma Chemical Co., St. Louis, MO, USA) was added to each well and the cells were incubated for 60 min at 37 °C to allow incorporation of BrdU into newly synthesized DNA during the S phase.

After incubation, the cells were washed with phosphate-buffered saline (PBS) and fixed with 70% ethanol. The fixed cells were rinsed with PBS, smeared onto glass slides, and maintained in PBS for 20 min. Endogenous peroxidase activity was quenched by incubation with hydrogen peroxide (H_2_O_2_) for 30 min in the dark, followed by three washes with PBS. The slides were then briefly incubated in distilled water and placed in an oven at 37 °C for 5 min. DNA denaturation was performed by incubating the slides in 4 N HCl for 30 min at 37 °C to expose incorporated BrdU. The slides were subsequently rinsed with distilled water and washed three times with PBS.

To minimize non-specific binding, the slides were incubated with a blocking solution and then exposed to the primary anti-BrdU antibody (BrdU Ab-4; Neomarkers, Sigma Chemical Co., St. Louis, MO, USA, 1848P 606F) for 1 h at room temperature in a humidified chamber. After washing with PBS, the slides were incubated with a biotinylated goat anti-mouse secondary antibody for 20 min, followed by streptavidin–peroxidase for an additional 20 min.

Immunoreactivity was visualized using an AEC substrate–chromogen system with a 20 min incubation in the dark. The slides were then rinsed with distilled water, counterstained with Mayer’s hematoxylin for 15 min, washed thoroughly with distilled water, and mounted using Ultramount mounting medium.

Cells incorporating BrdU were identified by red-stained nuclei, indicating cells in the S phase of the cell cycle. For quantitative evaluation, at least 100 cells were counted from three randomly selected microscopic fields on each slide, and the BrdU labeling index was calculated as the percentage of BrdU-positive cells relative to the total number of cells counted, according to previously described methods [49]. Parallel sections processed without primary antibody incubation were used to verify staining specificity. All experiments were performed in triplicate to ensure reproducibility.

### 4.5. Annexin V-FITC/PI Apoptosis Assay

The analysis was performed according to previously described protocols [50]. Apoptosis was quantitatively assessed using the Annexin V-FITC/Propidium Iodide (PI) apoptosis detection kit (BD Pharmingen™, San Diego, CA, USA). Following treatment, cells were harvested and processed according to the manufacturer’s protocol. Flow cytometric analysis enabled discrimination of viable (Annexin V^−^/PI^−^), early apoptotic (Annexin V^+^/PI^−^), and late apoptotic or necrotic (Annexin V^+^/PI^+^) cells. A minimum of 10,000 events per sample were acquired using a BD FACSCalibur flow cytometer (Becton, Dickinson and Company, San Jose, CA, USA). Data analysis was performed using CellQuest™ (BD Biosciences, San Jose, CA, USA) and FlowJo™ software v.11 (BD Biosciences, USA).

### 4.6. Cell Cycle Analysis

Cell cycle distribution was analyzed via flow cytometry according to previously described methods [50]. Briefly, cells were harvested and prepared as single-cell suspensions. The cells were fixed in ice-cold 70% ethanol and stored at −20 °C overnight. After fixation, the cells were washed with phosphate-buffered saline (PBS) and treated with RNase A (100 µg/mL) to remove RNA and ensure DNA-specific staining. The cells were then stained with propidium iodide (PI; 50 µg/mL) for 30 min at room temperature in the dark.

DNA content was analyzed using a BD FACSCalibur flow cytometer (BD Biosciences, USA), and at least 10,000 events were collected for each sample. The percentages of cells in the G_0_/G_1_, S, and G_2_/M phases of the cell cycle were determined using ModFit LT software v.3.3 (Verity Software House, Topsham, ME, USA). All experiments were performed in triplicate to ensure the reproducibility of the results.

### 4.7. Statistical Analysis

Statistical analyses were performed using GraphPad Prism software (version 8.0; GraphPad Software, San Diego, CA, USA). Data were expressed as mean ± standard deviation (SD). The normality of distribution was assessed using the Kolmogorov–Smirnov test. Differences among groups were evaluated using one-way ANOVA followed by Tukey’s post hoc test. A *p*-value < 0.05 was considered statistically significant.

## 5. Conclusions

This study demonstrates that resveratrol differentially modulates the cytotoxic, antiproliferative, and apoptotic responses of MCF-7 BC cells to adriamycin, paclitaxel, and cisplatin. The observed effects appear to be context-dependent, influenced by factors such as the type of chemotherapeutic agent, exposure concentration, and treatment duration evaluated in this study. In particular, the enhancement of apoptotic responses and suppression of cell proliferation in selected resveratrol–chemotherapy combinations suggest that resveratrol may influence treatment response beyond its intrinsic biological activity. Although derived from an in vitro model, these findings provide biologically relevant insights into the potential role of resveratrol as a complementary modulator of chemotherapy efficacy. Further preclinical and clinical investigations are warranted to better define the therapeutic contexts in which resveratrol-based combination strategies may be beneficial in BC management.

## Data Availability

The datasets generated and/or analyzed in the current study are available from the corresponding author upon reasonable request. All data supporting the findings of this study are included within the article.
